# Metals in *Callitriche cophocarpa* from small rivers with various levels of pollution in SW Poland

**DOI:** 10.1007/s11356-023-28372-5

**Published:** 2023-08-21

**Authors:** Przemysław Maksymowicz, Aleksandra Samecka-Cymerman, Adam Rajsz, Bronisław Wojtuń, Andrzej Rudecki, Maciej Lenarcik, Alexander J. Kempers

**Affiliations:** 1 Department of Ecology, Biogeochemistry and Environmental Protection, University of Wrocław, Ul. Kanonia 6/8, 50-328 Wrocław, Poland; 2 Institute for Water and Wetland Research, Department of Environmental Science, Radboud University, Huygens Building, Heijendaalseweg 135, Nijmegen, 6525 AJ The Netherlands

**Keywords:** Bioindication, Trace element, Aquatic macrophyte

## Abstract

The anthropogenic impact of metals on aquatic environments is a risk for biota, and thus their levels must be controlled. *Callitriche cophocarpa* Sendtn. belongs to a genus with a potential for accumulation of elevated metal levels. Thus, it may provide consolidated evidence of contamination. Therefore, the aim of this investigation was to determine Cd, Co, Cr, Cu, Fe, Mn, Ni, Pb and Zn in this species collected together with water and bottom sediments from rivers with various levels of pollution. Of these rivers, one less polluted and one more polluted was selected for the collection of *C. cophocarpa* for an experiment to compare its Cu and Zn concentration potential. Both metals were supplemented at concentrations 0.01, 0.02, 0.03, 0.05, 0.08 and 0.14 mg L^−1^ of Cu as CuSO4 × 5H2O and 0.4, 0.6, 0,9, 1,35, 2.03 and 3.04 mg L^−1^ of Zn as ZnSO_4_ × 7H_2_O, and in the binary design containing (mg·L^−1^) 0.01Cu + 0.4Zn, 0.02Cu + 0.6Zn, 0.03Cu + 0.9Zn, 0.05Cu + 1.4Zn, 0.08Cu + 2.03 Zn and 0.14Cu + 3.04Zn. The upper concentrations of Cr, Cu, Mn and Zn in *C. cophocarpa* shoots from both types of rivers as well as of Ni and Pb in shoots from more polluted rivers were higher than the values typical for toxicity thresholds with no visible harmful effects, which may indicate accumulation abilities of *C. cophocarpa* for these metals. Both roots and shoots of *C. cophocarpa* may be included in the group of macroconcentrators for bottom sediments with respect to Cd, Co, Cr, Cu, Fe, Mn, Ni and Zn and deconcentrators of Pb. Greater accumulation of most metals in roots than in shoots indicates their restricted mobility and translocation by *C. cophocarpa* to shoots. *C. cophocarpa* from the less polluted river and exposed to all experimental solutions contained significantly higher levels of Cu and Zn than that from the more polluted river exposed to identical experimental solutions. The plants collected from the more polluted river influenced by surplus of metals and living under chemical stress could probably limit further accumulation by developing a resistance mechanism. Cu and Zn contents in *C. cophocarpa* were higher when treated with separate metals than for binary treatment both in the more and less polluted river. Such research presenting the impact of a combination of metals could be important for understanding and explaining the interactions of these elements which may influence their bioavailability in nature as well as importance in the evaluation of the risk of environmental toxicity.

**Supplementary Information:**

The online version contains supplementary material available at 10.1007/s11356-023-28372-5.

## Introduction

Contamination of aquatic habitats is a crucial problem of ecological interest because of its harmful influence on living organisms, their biodiversity and water cleanliness (Mountouris et al. 2002). The anthropogenic impact may seriously affect the ecological status of surface waters by producing different categories of pollution and creating an environmental hazard for aquatic biota (Nábĕlková et al. 2004). Investigation concerning flora in running waters may give knowledge on the quality of such systems (Martinez and Shu-Nyamboli 2011). Especially aquatic macrophytes could be very useful in the bioindication but also in the bioremediation of toxic substances from polluted waters (Hansen et al. 2011; Harguinteguy et al. 2013). Dosnon-Olette et al. (2011) and Poklonov et al. (2021) presented these plants as a natural sink for contaminants able not only to remove them from the environment but also to metabolise them, thus protecting the ecosystem from their impact. Among xenobiotics especially metals are important water pollutants. These elements are non-degradable, and once released they may not be removed by natural processes of decomposition, and therefore accumulate in biotic and abiotic parts of the ecosystem (Miretzky et al. 2004; Khazheeva et al. 2005; Boudet et al. 2011). Macrophytes are able to diminish the level of metals by accumulating them in their tissues (Martinez and Shu-Nyamboli 2011; Bácsi et al. 2014; Novák et al. 2014 ). Thus, the metal content in aquatic vegetation is frequently much higher than in the environment (Miretzky et al. 2004). These plants may also serve as important identifiers of pollution sources (Berg and Steinnes 1997). Macrohydrophytes are immobile and constantly influenced by pollutants and thus may combine in their tissues variations in metal levels over time (Thiébaut et al. 2010). The load of metals concentrated by aquatic plants is crucially important for estimating the risk of environmental toxicity (Kabata-Pendias 2001; Nieder et al. 2004; Roy and Gunjan 2010). There is also evidence that some species of aquatic plants may hyperaccumulate metals and thus may be useful in remediation strategies (Brooks 1998; Favas et al. 2014). *Callitriche cophocarpa* Sendtn. belongs to a genus with a potential for accumulation of elevated metal levels. For instance, *C. stagnalis* was reported as a good accumulator of *U* reflecting the natural load of this element in mineralised areas (Favas et al. 2016). *C. palustris* has been shown as an accumulator of Cd, Cr, Cu, Fe, Ni, Pb and Zn in amounts corresponding to their concentrations in wastewaters (Poklonov et al. 2018). Furthermore *C. cophocarpa* is an efficient phytoremediator of Cd, Cr, Pb, Tl and Zn (Augustynowicz et al. 2010, 2016; Genchi et al. 2021). This ability is probably caused by the content of phenolic compounds and provides high protection against metal-induced oxidative stress (Augustynowicz et al. 2014a,b). The biomass of this species was proposed as a biosorbent for trivalent chromium (Kyzioł-Komosińska et al. 2018). Investigations into the influence of contamination on aquatic macrophytes usually concern single metals. However, these plants are usually impacted by their combination, which may mitigate or amplify effects (Ince et al. 1999; Pivetz 2001; Haiyan 2003). This study aimed to evaluate Cd, Co, Cr, Cu, Fe, Hg, Mn, Ni, Pb, Zn contents in water, bottom sediments and *C. cophocarpa* from 17 sites in small rivers with different contamination levels in SW Poland. A laboratory experiment was conducted to compare the influence of Cu and Zn (as prevailing metals in the chosen rives) after their single or combined application. The tested hypotheses were: binary Cu and Zn concentrations influencing *C. cophocarpa* may cause mitigating effects on the content of these metals in the species; *C. cophocarpa* collected from the more polluted river accumulates significantly more Cu and Zn than the same species from the less polluted river because it has developed adaptation to elevated levels of both elements.

Such research presenting the impact of a combination of metals could be important for understanding and explaining the interactions of these elements which may influence their bioavailability in nature as well as importance in the evaluation of the risk of environmental toxicity (Stewart and Malley 1999; Kabata-Pendias 2001; Roy and Gunjan 2010).

## Materials and methods

### Sampling design

**Fig. 1:**
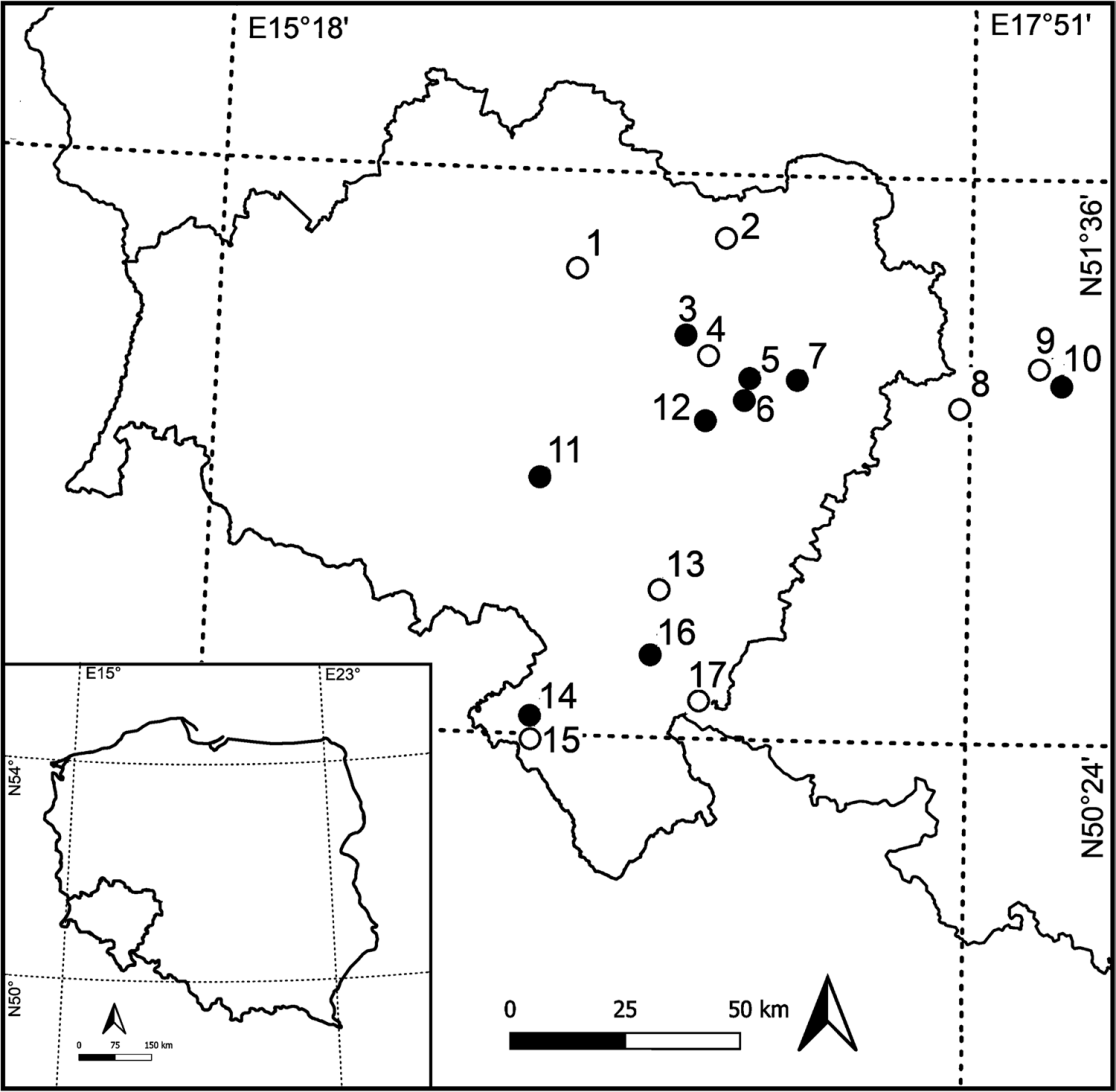
Location of the sampling sites; open circles: less polluted rivers, filled circles: more polluted rivers

Seventeen rivers in western Poland with various levels of pollution were selected (Fig. 1; ESM 1a,b). In each of these rivers, three sampling sites were selected randomly. Samples of water, sediment and *C. cophocarpa* in three replications were collected. Macrophytes were washed in water of their sampling sites to remove any attached material and then shoots and roots were separated.

### Water, bottom sediment and plant analysis

To determine total metal contents, water samples were acidified to pH ≤ 2 with ultra-pure HNO_3_ and filtered through 0.45-μm glass microfibre filters (Ladislas et al. 2012). Fresh sediments were used for potentiometric pHH_2_O determinations. Plants and bottom sediments were dried at 50 °C to constant weight. Plant samples were homogenised in a laboratory mill (IKA Labortechnik M20). Bottom sediments were homogenised using an agate mortar with a pestle after removing any coarse material with a 2-mm sieve. Plant-available Cd, Co, Cr, Cu, Fe, Mn, Ni, Pb and Zn in bottom sediments were assayed by extraction with 1 M ammonium acetate-EDTA (pH 4.65) for 30 min (10 g dry soil in 100 mL) (Cottenie et al. 1982). The total content of metals in sediments and plants (both shoots and roots) were evaluated using 300 mg of dry weight digested with 3 mL ultra-pure, 65% HNO_3_ and 2 mL ultra-pure 70% perchloric acid in a microwave oven (Speedwave Xpert Berghof). After dilution to 50 mL, the digests together with water samples were analysed for Fe, Mn and Zn using FAAS (Avanta PM from GBC) and Cd, Co, Cr, Cu, Ni and Pb using GFAAS (PinAAcle 900Z Perkin-Elmer Graphite Furnace Atomic Absorption Spectrometry). All the elements were determined against standards (Atomic Absorption Standard Solution from Sigma Chemical Co.), and blanks containing the same matrix as the samples and were subjected to the same procedure. All analyses were done in triplicate, and all results for plants were calculated on a dry weight basis. The accuracy of the methods applied for the determination of the elements in the samples was checked by the analysis of Chestnut Soil, Bainaimao and Bayan Obo, Neil Mongol in China GBW07402 (GSS-2) and Poaceae (mixture) IPE 952WEPAL Certified Reference Materials (Electronic Supplementary Material ESM 2a,b).

### Experimental setup

The experimental design was prepared based on investigations of Cegłowska et al. (2016). Samples of *C. cophocarpa* were collected from relatively unpolluted (no. 1) and potentially polluted (no. 3) river sites (Fig. 1; ESM 1a,b). Raised levels of Cu and Zn were found in the polluted rivers and, therefore, both elements were selected for further laboratory experiments (as single metals and binary combinations). The preliminary experiment proved, similarly to the results of Cegłowska et al. (2016), that Cu was more toxic than Zn. Therefore, we decided not to use equal concentrations of Cu and Zn in the study. The solutions of Cu and Zn were prepared as follows: 0.01, 0.02, 0.03, 0.05, 0.08 and 0.14 mg L^−1^ of Cu as CuSO4 × 5H_2_O and 0.4, 0.6, 0,9, 1,35, 2.03 and 3.04 mg L^−1^ of Zn as ZnSO4 × 7H_2_O were used. In addition, mixed solutions of both metals were prepared containing (mg·L^−1^) 0.01Cu + 0.4Zn, 0.02Cu + 0.6Zn, 0.03Cu + 0.9Zn, 0.05Cu + 1.4Zn, 0.08Cu + 2.03 Zn and 0.14Cu + 3.04Zn (Cegłowska et al. 2016). After 3 days of acclimatisation (Buta et al. 2014) in a 0.5% Hoagland nutrient solution at pH 6.0 (adjusted with 0.1 M HCl or NaOH) (Nyquist and Greger 2007; Sun et al. 2011), plants were cultivated in 1-L beakers covered with transparent plastic film at a solution temperature of about 21 °C according to Fritioff et al. (2005) and Cegłowska et al. (2016). Ten pieces of 10 cm-long healthy-looking upper vegetative parts of *C. cophocarpa* shoots (Temel 2005) were placed in the 0.5% Hoagland solution with different concentrations of Cu, Zn or Cu + Zn. Control, with the 0.5% Hoagland solution and plants, and negative control, with the 0.5% Hoagland solution and the metals without plants, was prepared. The beakers were arranged randomly in five replicates for each treatment. Each group consisted of 8 sampling points (including two controls) × 3 metal combinations × 5 replicates = 120 (Cegłowska et al. 2016). The plants were cultivated in a glasshouse under daylight for 7 days (Mal et al. 2002; Wang et al. 2009). At the end of the experiment, the changes in Cu and/or Zn concentrations in the plants were determined using the methods presented above.

### Statistical analysis

One-way ANOVA (*p* < 0.05) on Box-Cox transformed data was applied to calculate the differences among the metal concentrations in water, sediments and *C. cophocarpa* between the investigated rivers (Balabanova et al. 2017). Normality of the features was controlled by Shapiro–Wilk’s *W* test and homogeneity of variances was evaluated by the Brown-Forsythe test. The sites were divided into more and less polluted based on standardised Cu and Zn concentrations. The mean of the sum of the standardised values of both elements was estimated. The decisive criterion established was the median value for standardised data equal to − 0.21. The less polluted sites were no 1, 2, 4, 8, 9, 10, 15, 17 and the more polluted ones were no 3, 5, 6, 7, 11, 12, 13, 14 and 16 (Fig. 1; ESM 1a,b). Metal concentrations in *C. cophocarpa* shoots and roots between less and more polluted rivers as well as those between shoots and roots were compared with the Wilcoxon signed-rank test (*P* < 0.05). The post hoc Tukey test was used to compare Cu or Zn contents in experimental *C. cophocarpa* when Cu, Zn and Cu + Zn were added (Zar 1999). Element contents in experimental *C. cophocarpa* from less and more polluted rivers and with added metals (Cu + Zn versus Zn and Cu + Zn versus Cu) were compared with the *t*-test (Zar 1999). For evaluation of contamination of bottom sediments of *C. cophocarpa*, the contamination factor CF, contamination degree CD and pollution load index PLI (Håkanson 1980, Tomlinson et al. 1980) were calculated. The contamination factor for a selected metal CF = *C*_*m*_*/C*_0_, where *C*_*m*_ is the metal content in sediment 0, and *C*_0_ is the geochemical background value according to Bojakowska (2001). The classes of sediment pollution based on contamination factor are as follows: CF < 1 low contaminated, 1 ≤ CF < 3 moderately contaminated, 3 ≤ CF < 6 significantly contaminated, and 6 ≤ CF heavily contaminated. Degree of contamination CD was calculated as the sum of contamination factors determined for individual metals, with the following classification range: CD < 8 low degree of contamination, 8 ≤ CD < 16 moderate degree of contamination, 16 ≤ CD < 32 considerable degree of contamination, 32 < CD very high degree of contamination indicating serious anthropogenic pollution. The pollution load index was calculated of all multiplicated CF values for analysed metals as follows: PLI = (*CF*_*m*1_ × *CF*_*m*2_ × *CF*_*m*3_ × … × *CF*_*n*_)^1/*n*^. The general toxicity condition of the sample: PLI ≤ 1 shows baseline levels of pollutants while its growth to 1 < PLI shows progressive deterioration of aquatic ecosystem quality (Håkanson^1980^; Tomlinson et al. 1980).

The bioaccumulation factor was established as a ratio of the metal content in shoots with leaves or roots to the metal that in water (BFw) or a plant-available metal in bottom sediments (BFs) (Szczepaniak and Biziak 2003). The transfer factor (TF) was calculated as a ratio of metal contents in shoots with leaves to roots.

All calculations were done with StatSoft 13 software (StatSoft (2017).

## Results and discussion

### Water, bottom sediments and plants

ANOVA proved significant differences among metal contents in water, sediments and plant samples between the sites. Cr, Cu, Ni, Pb and Zn concentrations in the water of less polluted rivers were significantly lower than in the more polluted rivers (ESM 3). Metal concentrations in the water of both types of rivers were lower than the values typical for waters with purity grade 1 (ESM 3) but higher than in unpolluted waters where *C. cophocarpa* occurred (Augustynowicz et al. 2014b). It should be taken into account that water analysis is much less precise than plant analysis because it reflects only the current contamination status (Harguinteguy et al. 2013). Macrophytes are not mobile, and thus constantly exposed to pollution and cannot avoid metals which affect their habitats. Therefore, these plants may be used as indicators of the combination of chemical stressors integrated in time (Miretzky et al. 2004; Thiébaut et al. 2010; Rajfur et al. 2010; Pereira et al. 2012; Bytyqi et al. 2020). The total concentrations of Cd, Co, Cr, Cu, Ni and Zn in bottom sediments of less polluted rivers were significantly lower than in more polluted rivers (ESM 4). Median values of total elements in bottom sediments of both less and more polluted rivers were lower than recommended for sediments with purity grade 1 (Bojakowska 2001; Michalec 2012). However, the upper levels of total Co, Cr, Ni, Pb and Zn in both types of rivers were higher than this threshold, which may indicate pollution of some sites with these elements (Michalec 2012). Median values of the contamination factor CF for bottom sediments of less polluted rivers showing mostly a low contamination were lower than CF for polluted ones showing mostly moderate contamination. The exception was significant contamination with Cr and heavy contamination with Ni of bottom sediments of polluted rivers (ESM 5). Median values of the degree of contamination (CD) for bottom sediments of less polluted rivers were within the scope of low degree of contamination and for polluted ones were within the scope of considerable degree of contamination (ESM 5). Median values of the pollution load index (PLI) for bottom sediments of less polluted rivers were within baseline of pollution and of polluted rivers within progressive deterioration of river quality (ESM 5). Plant-available concentrations of all elements except for Fe and Mn (which did not quality differ) in the bottom sediments of less polluted rivers were significantly lower than in more polluted rivers (ESM 6). Concentrations of all elements except for Fe and Mn (which did not differ) in *C. cophocarpa* shoots of less polluted rivers were significantly lower than in more polluted rivers (Table 1). The upper concentrations of Cr, Cu, Mn and Zn in shoots from both types of rivers as well as Ni in shoots from more polluted rivers were higher than the values typical for toxicity thresholds (Table 1) in plants (Kabata-Pendias 2001) with no visible harmful effects. This may indicate a high metal accumulation capacity of *C. cophocarpa* for these metals. Median values of Cu, Fe, Mn, Ni and Zn concentrations in shoots of this species from less and more polluted rivers were also higher than the average content for terrestrial plants presented (in mg kg^−1^) by Kabata-Pendias (2001): Cu < 5, Fe 130–350, Mn 15–160, Ni < 1, and Zn 15–30. As a submerged macrophyte, *C. cophocarpa* takes up metals from sediments not only with roots but also from water through stems and leaves (Dhir et al. 2009). Submerged macrophytes have greater surface areas in comparison with non-submerged ones, which makes them suitable for the bioaccumulation of metals (Sharmila and Saradhi 2009; Cai et al. 2018). Thus, the whole surface of *C. cophocarpa* is probably important for a mechanism of contaminant removal (Nyquist and Greger 2007). There is a general rule that aquatic plants contain many times as high metal concentrations as ambient water (Cordeiro et al. 2016). Augustynowicz et al. (2013) report an unusual accumulation potential of this species for Cr. Owing to the high level of the phenolic compounds with very high scavenging activity protecting *C. cophocarpa* from reactive oxygen species, this plant can probably accumulate also other metals (Augustynowicz et al. 2014a).

**Table 1:** Minimum, maximum and median of metal concentrations (mg·kg^−1^) in shoots of *Callitriche cophocarpa. p* for the *U* Mann–Whitney test comparing clean and polluted sites. The data in the column Threshold are environmental threshold toxicity limits of metal ions established for plants by Kabata-Pendias (Kabata-Pendias 2001). NS, not significant; BDL, below detection limit: mg·kg^−1^ Pb < 0.025

	Clean			Polluted				
	Minimum	Maximum	Median	Minimum	Maximum	Median	*p*	Threshold
Cd	0.02	1.1	0.08	0.03	1.1	0.2	< 0.01	5–30
Co	0.6	4.3	3.5	0.5	14	4.2	< 0.05	15–50
Cr	0.2	7.9	1.3	0.2	22	5.4	< 0.01	5–30
Cu	4.3	17	9.5	11	27	21	< 0.01	15–20
Fe	1102	19,886	4583	751	7392	3585	NS	
Mn	98	9862	5361	1452	11,688	5413	NS	400–1000
Ni	1.1	4.7	2.7	3.9	17	6.5	< 0.01	10–100
Pb	BDL	1.4	0.5	0.5	11	2.0	< 0.01	30–300
Zn	53	115	73	71	247	137	< 0.01	100–400

Concentrations of all elements except for Cd, Fe and Mn in *C. cophocarpa* roots (which did not differ) of less polluted rivers were significantly lower than in more polluted rivers (Table 2). *C. cophocarpa* roots from less and more polluted sites accumulated significantly more metals than shoots (ESM 7 and 8). The only exception was the concentration of Cu in this species from less polluted sites which was higher in shoots than in roots. Ni and Zn concentrations in *C. cophocarpa* from less polluted sites as well as those of Cr and Cu in this species from more polluted sites did not differ between shoots and roots. Plants usually protect their photosynthetic tissues from metals and restrict their accumulation to roots (Koblar et al. 2015). This phenomenon is probably caused by the fact that Cu and Zn are essential elements necessary for the proper growth of the examined species (Olivares et al. 2009; Nabi 2021). According to Augustynowicz et al. (2020) *Callitriche* absorbs Cr through the shoots. The concentration of ions in aquatic plants is usually at some equilibrium between uptake and leakage of accumulated metals back into water (Nyquist and Greger 2007). However, some species develop protection from leakage of essential metals. For instance, *Elodea canadensis*, another submerged species, produces antioxidant enzymes providing defence against increased concentrations of these elements. Cu and Zn loaded in plant tissues are incorporated in the apoplastic zone where they are protected from easy leakage out (Nyquist and Greger 2007). In spite of the fact that Cu and Zn were accumulated in tissues of *E. canadensis*, this species was able to further absorb metals (Nyquist and Greger 2007; Ladislas et al. 2012). A similar mechanism might have been developed also by *C. cophocarpa.*

**Table 2:** Minimum, maximum and median of metal concentrations (mg·kg^−1^) in roots of *Callitriche cophocarpa. p* for the *U* Mann–Whitney test comparing clean and polluted sites. NS, not significant; BDL, below detection limit: mg·kg^−1^ Ni < 0.05 and Pb < 0.001

	Clean			Polluted			
	Minimum	Maximum	Median	Minimum	Maximum	Median	*p*
Cd	0.1	2.9	0.2	0.05	1.8	0.3	NS
Co	0.7	16	11	4.6	44	13	< 0.05
Cr	1.1	9.1	2.7	2.1	43	3.7	< 0.05
Cu	2.0	16	7.3	9.5	29	20	< 0.01
Fe	1120	87,960	8958	1922	17,824	6445	NS
Mn	455	23,515	16,165	2295	47,975	9685	NS
Ni	BDL	21	2.8	6.4	22	11	< 0.01
Pb	BDL	8.7	0.7	1.3	28	5.8	< 0.01
Zn	40	213	66	75	347	193	< 0.01

### Element relocation

For the determination of metal relocation from water and bottom sediments to plants, bioaccumulation factors (BFw and BFs, respectively) were estimated (ESM 9–10). In plants able to absorb metals from the substrate and further transfer them to the upper parts, this factor has values higher than one (Galal and Shehata 2015). Both factors are important parameters for understanding the availability of metals to plants (Angelone et al. 1993). Median values of BF from water to both roots and shoots of more and less polluted rivers were much higher than 1 only for Mn, followed by Fe. Thus, these metals were the most intensively accumulated by *C. cophocarpa*. A similar trend was obtained for *Elodea canadensis*, a different aquatic macrophyte accumulating also the highest amounts of Fe (Busuoic et al. 2012).

Median values of BF from bottom sediments to both roots and shoots of more and less polluted rivers were higher than 1 for all metals except Pb (Tables 3 and 4). Khazheeva et al. (2005) suggest that the BF from sediments is more valuable than that from water. According to the scale of BFs proposed by Khazheeva et al. (2005) for metal concentrations in bottom sediments, both roots and shoots of *C. cophocarpa* may be included in the group of macroconcentrators (BFs > 2) for Cd, Co, Cr, Cu, Fe, Mn, Ni and Zn and deconcentrators for Pb (BFs < 1). Probably, this species restricts accumulation of Pb as a metal not essential for plants, toxic even in very low concentrations (Hadi and Aziz 2015). Roots and shoots were accumulating metals in a similar order: roots from less polluted rivers Cr > Zn > Mn > Fe > Co > Ni > Cu/Cd > Pb; roots from more polluted rivers Cr > Mn > Zn > Co > Fe > Ni > Cu > Cd > Pb; shoots from less polluted rivers Cr > Zn > Mn > Fe > Cu > Co > Ni > Cd > Pb; and shoots from more polluted rivers Cr > Zn > Mn > Fe > Co > Cu > Ni > Cd > Pb. The most accumulated metals from sediments were Cr, Zn and Mn, while Pb was the least accumulated metal. These results are consistent with the studies of other authors that *C. cophocarpa* is an efficient phytoremediator of Cr and Zn (Augustynowicz et al. 2010, 2014a,c, 2016; Genchi et al. 2021). However, the same authors recommend this species also as an efficient Pb accumulator. The other discrepancy is the statement of Augustynowicz et al. (2020) that *C. cophocarpa* absorbs Cr by shoots with very low concentration factors for roots.

**Table 3:** Minimum, maximum, and median of the bioaccumulation factor (ratio of metal concentration in shoots to plant-available metal in bottom sediments)

	Clean			Polluted		
	Minimum	Maximum	Median	Minimum	Maximum	Median
Cd	0.1	4.6	1.0	0.2	5.6	0.8
Co	1.1	9.6	2.1	1.3	11	3.8
Cr	2.8	129	29	1.3	82	48
Cu	0.9	12	2.9	0.6	21	3.0
Fe	0.5	21	6.1	1.2	31	4.2
Mn	1.2	82	8.3	3.8	143	10
Ni	0.6	14	1.9	0.6	14	2.8
Pb	0.001	0.3	0.1	0.07	2.5	0.3
Zn	4.1	129	17	6.9	76	16

**Table 4:** Minimum, maximum and median of the bioaccumulation factor (ratio of metal concentration in roots to plant-available metal in bottom sediments) for metals in *Callitriche cophocarpa* from clean and polluted sites

	Clean			Polluted		
	Minimum	Maximum	Median	Minimum	Maximum	Median
Cd	0.5	35	2.1	0.4	9.0	1.2
Co	1.8	33	6.7	2.7	35	9.6
Cr	14	154	51	11	202	31
Cu	0.9	8.5	2.1	0.5	13	2.8
Fe	1.2	98	9.6	3.2	45	8.8
Mn	4.4	24	14	11	36	19
Ni	0.7	34	3.2	1.1	30	3.3
Pb	0.04	2.3	0.4	0.2	16	1.0
Zn	4.6	27	15	9.5	36	11

Transfer of metals from roots to shoots was calculated to involve internal transport of these elements in *C. cophocarpa* (Table 5)*.* The obtained median values indicate that metals accumulated by this species were mostly retained in roots. Transport from roots to shoots with TF > 1 occurred only for Cu from clean sites and Cr in polluted sites. Greater accumulation of most metals in roots than in shoots indicates their restricted mobility and translocation once absorbed by *C. cophocarpa*. Such a mechanism makes it possible for plants to survive in polluted habitats. Kachenko et al. (2007) recognise sequestration of metals in roots as an important mechanism of metal tolerance (Kachenko et al. 2007), and thus various plant species accumulate non-essential metals mostly in roots (MacFarlane and Burchett 2000; Tyler 2004; Romero-Hernández et al. 2017). Buta et al. (2014) also observed higher concentrations of metals in *Eichhornia crassipes* roots than in leaves. The obtained results are in contradiction with the statement of Madsen and Cedergreen (2002) that roots of submerged aquatic plants have an anchoring function rather than accumulate elements. Further investigation is necessary to solve these problems.

**Table 5:** Minimum, maximum and median of the transfer factor (ratio of metal concentration in shoots to roots) for metals in *Callitriche cophocarpa* from clean and polluted sites

	Clean			Polluted		
	Minimum	Maximum	Median	Minimum	Maximum	Median
Cd	0.1	0.8	0.3	0.3	1.0	0.5
Co	0.2	1.2	0.4	0.02	0.9	0.4
Cr	0.02	1.4	0.6	0.1	2.5	1.2
Cu	1.0	2.0	1.2	0.8	1.3	1.0
Fe	0.2	1.0	0.4	0.2	1.0	0.5
Mn	0.2	2.2	0.4	0.2	1.3	0.5
Ni	0.2	1.4	0.6	0.4	0.9	0.6
Pb	0.02	2.4	0.3	0.1	1.9	0.3
Zn	0.5	1.5	1.0	0.6	1.0	0.8

### Laboratory experiment: accumulation of Cu and Zn by *C. cophocarpa*

**Fig. 2:**
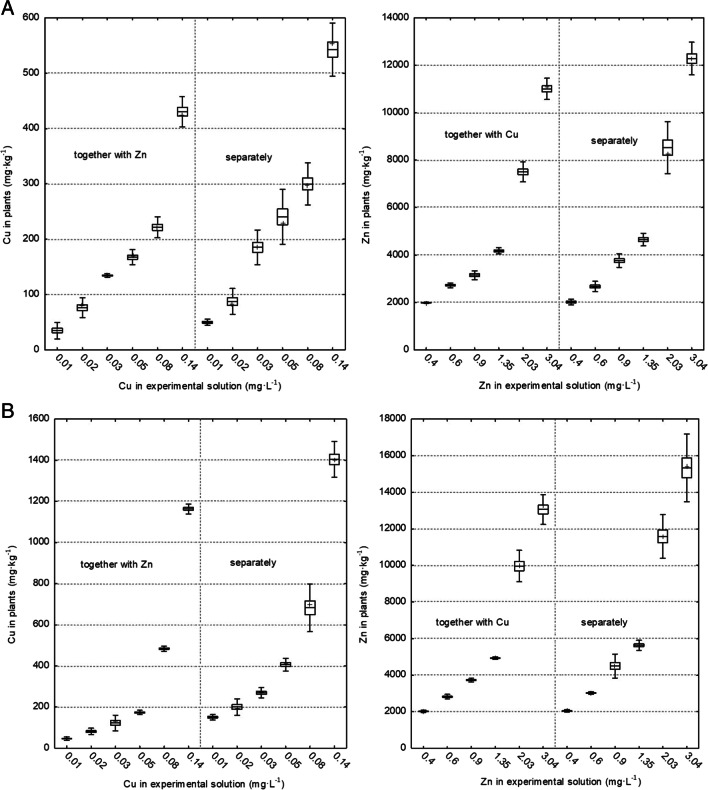
**A** Concentration (mg∙L^−1^) of Cu and Zn in plants from the more polluted river influenced by Cu added (0.01, 0.02, 0.03, 0.05, 0.08 and 0.14), and together with Zn as 0.4Zn + 0.01Cu, 0.6Zn + 0.02Cu, 0.9Zn + 0.03Cu, 1.4Zn + 0.05Cu, 2.03Zn + 0.08Cu and 3.04Zn + 0.14Cu; and influenced by Zn added (0.4, 0.6, 0.9, 1.4, 2.03 and 3.04); line = mean, box = mean ± SE (standard error), whisker = mean ± 0.95 confidence interval. **B** Concentration (mg∙L^−1^) of Cu and Zn in plants from the less polluted river influenced by Cu added (0.01, 0.02, 0.03, 0.05, 0.08 and 0.14), and together with Zn as 0.4Zn + 0.01Cu, 0.6Zn + 0.02Cu, 0.9Zn + 0.03Cu, 1.4Zn + 0.05Cu, 2.03Zn + 0.08Cu and 3.04Zn + 0.14Cu; and influenced by Zn added (0.4, 0.6, 0.9, 1.4, 2.03 and 3.04); line = mean, box = mean ± SE (standard error), whisker = mean ± 0.95 confidence interval

At the end of the experiment, the content of Cu and Zn in both controls did not differ from that on day zero (maximum difference of 1.2%). On day 7, C. *cophocarpa* from the less polluted river and exposed to all experimental solutions contained significantly higher levels of Cu and Zn than that from the more polluted river exposed to identical experimental solutions (Fig. 2A, B). The plants collected from the more polluted river influenced by surplus of metals and living under chemical stress could probably limit further accumulation by developing a resistance mechanism (Kabata-Pendias 2001; Mehes-Smith et al. 2013). Such mechanism of plants to metals is complex and varies depending on the type of metal that plants face and the molecular characteristic of the species. All metals appear to have different mechanisms of toxicity which makes it difficult to hypothesise a common resistance mechanism against all metals and metalloids (Kalaivanan and Ganeshamurthy 2016). Metal-resistant plants utilise various strategies, including blocking metal absorption, extracellular and cytoplasmic complexation, and chelation, which requires the activation of defence responses, such as antioxidant enzyme expression, to prevent or repair oxidative stress-induced damage (Yu et al. 2019). Chelation is the process of binding metals to organic molecules such as phytochelatins (Callahan et al. 2006; Raychaudhuri et al. 2021). Some plants limit the inflow of excess of toxic compounds by sequestering metals in vacuoles, binding them to cell walls, or secreting them out, while others adapt by changing their architecture (Ghori et al. 2019; Yan et al. 2020). *C. cophocarpa* uses trichomes to adsorb, e.g. chromium and reduce it to less toxic forms. These epidermal glands are able to secrete substances into the external environment and accumulate cations of some metals, regulating their flow (Lavid et al. 2001; Augustynowicz et al. 2014b, 2014c). It may be the case that *C. cophocarpa* unlike *E. nuttallii* did not exhibit adaptation of its energy sources upon exposure to metals (Larras et al. 2013). However, a precise solution to this problem needs further investigation.

The species from both the more and the less polluted river accumulated significantly lower concentrations of Cu for all combinations and Zn for 0.03Cu + 0.9Zn, 0.05Cu + 1.4Zn, 0.08Cu + 2.03Zn and 0.14Cu + 3.04Zn combinations applied in the binary fashion than when applied as a single metal reaching a level of 1450 mg kg^−1^ Cu and 16,210 mg kg^−1^ Zn (from the less polluted river) with no visible symptoms of harmful effects (Fig. 2A, B). Absence of toxicity symptoms may be caused by accumulation of metals in cell walls (Larras et al. 2013) or/and by the content of phenolic compounds which provides a high protection against metal-induced oxidative stress (Augustynowicz et al. 2014a,b). These values are much higher than in the experiment of Cegłowska et al. (2016) where *Elodea canadensis* accumulated up to 63 mg kg^−1^ Cu and up to 1112 mg kg^−1^ Zn. These results are in agreement with the investigation of Luo and Rimmer (1995) that addition of Cu increases the toxicity of the added zinc. Kabata-Pendias (2001) reports that the interaction between Cu and Zn is antagonistic which means that the presence of one metal may decrease the accumulation of the other. However, these results are in contradiction with the investigation of Buta et. (2014), reporting that aquatic macrophytes were much less effective accumulators of Cu and Zn applied as single metals than in a multimetallic system. Upadhyay et al. (2011) and Buta et al. (2014) believe that macrophytes influenced by the toxic effect of metals usually require more Zn than those living in clean water because Zn is important for the antioxidant protection system, among other things (Upadhyay et al. 2011; Buta et al. 2014). Results of this investigation do not confirm this defensive role of Zn for *C. cophocarpa* because the Zn content in this species was higher in a single-treated culture than in Cu + Zn applications. Robson (1993) reports suppression or decrease in Cu uptake by Zn but not the opposite (decrease of Zn absorption by Cu) in the same experiment. These discrepancies may be explained by the fact that interactions between metals depend on their mutual ratios as well as quantitative proportions (Kabata-Pendias 2001; Li et al. 2009). Robson (1993) shows lack of interactions between Cu and Zn in water solutions and explains this phenomenon by the much lower influence of complex formation on the reactions of both ions in such solutions. The definitive clarification of these relations needs additional investigation.

## Conclusions

1. *C. cophocarpa* accumulated more Cu and Zn when applied as a single metal and less in a combined form. Probably antagonistic interactions between Cu and Zn may cause a decrease in the accumulation of one metal in the presence of the other.
2. *C. cophocarpa* collected from a more polluted riverliving under chemical stress could probably limit further accumulation of metals by developing a resistance mechanism, thus accumulating significantly less Cu and Zn than the same species from a less polluted river.

## Supplementary Information

Below is the link to the electronic supplementary material.

Supplementary file1 (PDF 402 KB)

Supplementary file2 (PDF 22 KB)

Supplementary file3 (PDF 230 KB)

## Data Availability

The datasets used and/or analysed during the current study are available from the corresponding author on reasonable request.
