# Supplementary material for: Metals in Callitriche cophocarpa from small rivers with various levels of pollution in SW Poland
Source: Environ Sci Pollut Res Int. 2023 Aug 21;30(43):97888–99. doi: 10.1007/s11356-023-28372-5 (PMC10495474; doi:10.1007/s11356-023-28372-5)

ESM 1a Detailed locations of the sampling sites 1-10 as indicated in the Figure 1; open circles: less polluted rivers, full circles: more polluted rivers

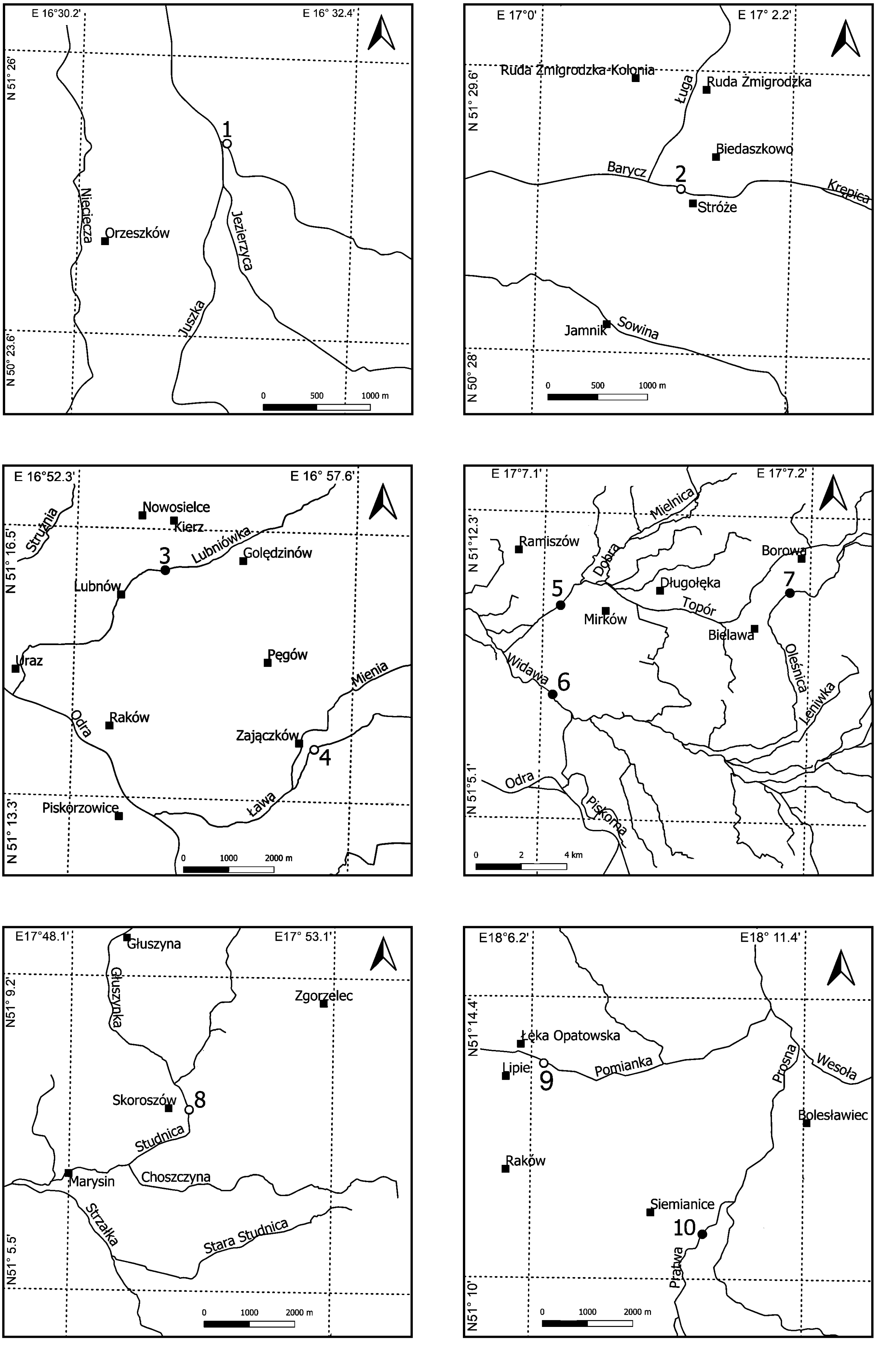

ESM 1b Detailed locations of the sampling sites 11-17 as indicated in the Figure 1; open circles: less polluted rivers, full circles: more polluted rivers

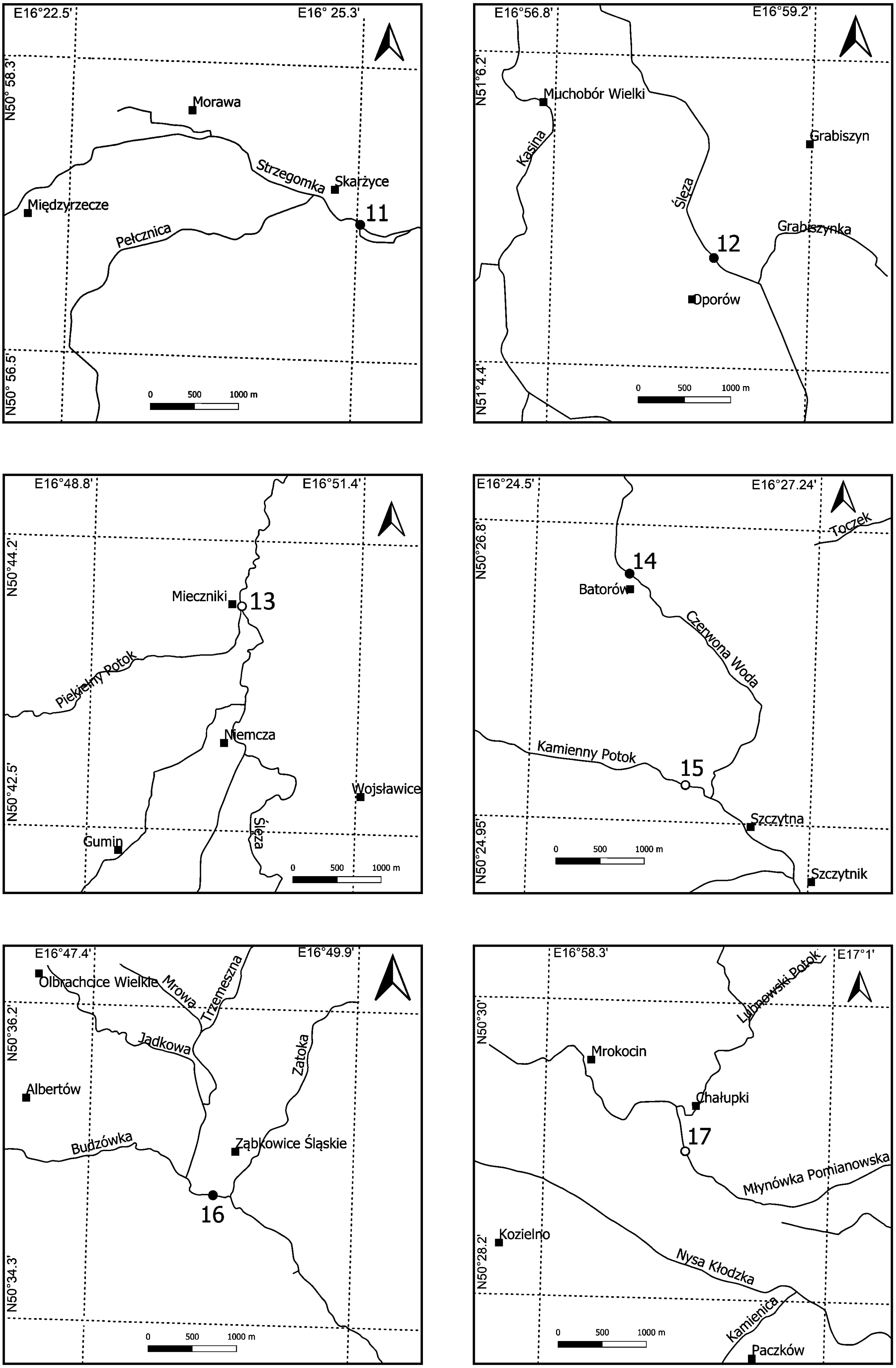

Supplement: Supplementary file 1 — Supplementary file1 (PDF 402 KB) [file 11356_2023_28372_MOESM1_ESM.pdf]
