# Supplementary material for: Metals in Callitriche cophocarpa from small rivers with various levels of pollution in SW Poland
Source: Environ Sci Pollut Res Int. 2023 Aug 21;30(43):97888–99. doi: 10.1007/s11356-023-28372-5 (PMC10495474; doi:10.1007/s11356-023-28372-5)
Supplement: Supplementary file 2 — Supplementary file2 (PDF 22 KB) [file 11356_2023_28372_MOESM2_ESM.pdf]

ESM 2a Analysis of Chestnut Soil, Bainaimao and Bayan Obo, Neil Mongol in China GBW07402 (GSS-2) certified reference material

Element	Certified	Found/Recovered	Recovery	CV
	$\mu g \cdot g^{-1}$		%	
Cd	0.071±0.014	0.073±0.014	102.82	19.2
Co	9.09±0.15	8.99±0.15	98.90	1.7
Cr	47±4	49±4.0	104.26	8.2
Cu	16.3±0.90	16.5±0.9	101.23	5.5
Fe	40900±0.07	41500±0.61	101.47	1.5
Mn	510±16	508±16	99.61	3.1
Ni	19.4±1.3	19.7±1.3	101.55	6.6
Pb	20±3	20.5±3.0	102.50	14.6
Zn	42±3	42.4±3.0	100.95	7.1

ESM 2b Analysis of Poaceae (mixture) IPE 952WEPAL Certified Reference Material

Element	Certified	Found/Recovered	Recovery	CV
	$\mu g \cdot g^{-1}$		%	
Cd	0.16±0.08	0.19±0.08	118.75	42.1
Co	2.36±0.01	2.39±0.0.01	101.27	0.4
Cr	2.36±0.01	2.39±0.01	101.27	0.4
Cu	9.97±0.13	9.49±0.13	95.19	1.4
Fe	40900±0.07	41500±0.61	101.47	1.5
Mn	510±16	508±16	99.61	3.1
Ni	19.4±1.3	19.7±1.3	101.55	6.6
Pb	20±3	20.5±3.0	102.50	14.6
Zn	42±3	42.4±3.0	100.95	7.1
