# Supplementary material for: Metals in Callitriche cophocarpa from small rivers with various levels of pollution in SW Poland
Source: Environ Sci Pollut Res Int. 2023 Aug 21;30(43):97888–99. doi: 10.1007/s11356-023-28372-5 (PMC10495474; doi:10.1007/s11356-023-28372-5)
Supplement: Supplementary file 3 — Supplementary file3 (PDF 230 KB) [file 11356_2023_28372_MOESM3_ESM.pdf]

ESM 3. Minimum, maximum and median of metal concentrations (µg·L^{-1}) in the water of rivers with *Callitriche cophocarpa*. P for the U Mann-Whitney test comparing clean and polluted sites. The data in the column Unpolluted are from clean sites of this species investigated by Augustynowicz et al. (2014b). In the column Threshold, environmental threshold limits of metal ions established for surface water with purity grade 1 are listed (Polish Journal of Laws, 2004). NS=not significant; BDL=below detection limit (µg·L^{-1}): Cd<0.000015, Cr<0.00005, Cu<0.0001, Pb<0.00025 and Zn <0.0005

	Clean			Polluted					
	Minimum	Maximum	Median	Minimum	Maximum	Median	p	Unpolluted	Threshold
Cd	BDL	0.2	0.02	BDL	0.3	0.05	NS	<0.0005	≤0,5
Cr	BDL	0.6	0.08	0.03	1.9	0.5	<0.01	<0.001	≤50
Cu	BDL	3.1	0.0001	BDL	6.4	1.0	<0.05	<0.0005	≤20
Fe	0.1	4.3	0.6	0.1	2.3	0.6	NS	0.02	≤100
Mn	0.01	0.8	0.2	0.05	0.2	0.1	NS	4.2	
Ni	0.16	2.6	1.4	0.9	5.6	3.2	<0.01	<0.0001	≤10
Pb	BDL	0.4	0.003	BDL	2.1	0.4	<0.01	<0.0005	≤10
Zn	BDL	4.2	1.3	1.6	16	3.2	<0.01	0.002	≤300

ESM 4. Minimum, maximum and median of total metal concentrations (mg·kg^{-1}) and pH in bottom sediments in rivers with *Callitriche cophocarpa*. P for the U Mann-Whitney test comparing clean and polluted sites. In the column Threshold, environmental threshold limits of metal ions established for sediments with purity grade 1 are listed (Bojakowska 2001; Michalec, 2012). NS=not significant; BDL=below detection limit (mg·kg^{-1}): Cd<0.000015, Co<0.01, Mn<0,15, Ni<0.05, Pb<0.00025 and Zn <0.0005

	Clean			Polluted				
	Minimum	Maximum	Median	Minimum	Maximum	Median	p	Threshold
Cd	BDL	0.7	0.2	0.1	2.1	0.5	<0.05	<1.0
Co	BDL	11	2.2	0.9	47	4.7	<0.01	<10
Cr	1.4	54	8.4	4.1	99	16	<0.01	<20
Cu	0.4	8.4	2.3	1.1	20	5.4	<0.01	<20
Fe	1272	45241	25026	2528	37918	19288	NS	
Mn	BDL	5020	1616	120	2920	1073	NS	
Ni	BDL	52	5.5	0.2	154	23	<0.05	<30
Pb	BDL	90	38	1.6	113	33	NS	<50
Zn	BDL	242	94	28	878	193	<0.05	<200
pH	6.3	7.6	6.9	5.5	7.9	6.8	NS	

ESM 5. Minimum, maximum and median of contamination factor CF, contamination degree CD, and pollution load index PLI (Håkanson 1980, Tomlison et al. 1980) for total metal concentrations in bottom sediments of *Callitriche cophocarpa* clean and polluted sites

	Clean			Polluted		
	Minimum	Maximum	Median	Minimum	Maximum	Median
CF_{Cd}	<0.01	1.4	0.4	0.2	4.2	1.0
CF_{Co}	<0.01	3.7	0.8	0. 3	16	1.6
CF_{Cr}	0.23	9.0	1.4	0.7	17	2.7
CF_{Cu}	0.06	1.2	0.3	0.2	2.9	0.8
CF_{Ni}	0.01	8.7	0.9	0.03	26	3.8
CF_{Pb}	<0.01	6.0	2.5	0.1	7.5	2.2
CF_{Zn}	<0.01	3.3	1.3	0.4	12	2.6
CD	0.31	33	8.0	2.2	75	16
PLI	0.01	3.5	0.9	0.2	8.3	1.4

ESM 6. Minimum, maximum and median of plant-available metal concentrations ($mg \cdot kg^{-1}$) in bottom sediments in rivers with *Callitriche cophocarpa.* P for the U Mann-Whitney test comparing clean and polluted sites. NS=not significant.

	Clean			Polluted			
	Minimum	Maximum	Median	Minimum	Maximum	Median	p
Cd	0.003	0.4	0.1	0.04	0.5	0,2	<0.01
Co	0.01	2.1	0.4	0.2	5.4	1.3	<0.01
Cr	0.02	0.1	0.06	0.1	0.3	0.2	<0.01
Cu	0.1	18	1.4	1.1	39	6.2	<0.01
Fe	76	2115	836	116	1675	1468	NS
Mn	4.7	1734	298	10	1327	345	NS
Ni	0.02	5.0	0.4	0.2	13	2.6	<0.01
Pb	0.1	17	4.7	1.0	30	6.3	<0.05
Zn	0.5	14	4.2	2,5	29	12	<0.01

ESM 7. Minimum, maximum and median of metal concentrations ($mg \cdot kg^{-1}$) in shoots and roots of *Callitriche cophocarpa* from less polluted rivers. P for the Wilcoxon test for comparison of metal concentrations between shoots with roots. NS=not significant

	Shoots			Roots			
	Minimum	Maximum	Median	Minimum	Maximum	Median	p
Cd	0.02	1.1	0.1	0.1	2.9	0.2	<0.01
Co	0.6	4.3	3.5	0.7	16	11	<0.01
Cr	0.2	7.9	1.3	1.1	9.1	2.6	<0.01
Cu	4.3	17	9.5	2.5	16	7.3	<0.01
Fe	1102	19886	4583	1120	87960	8958	<0.01
Mn	98	9862	5361	415	23515	16165	<0.01
Ni	1.1	4.7	2.7	0.03	21	2.8	NS
Pb	0.01	1.4	0.5	0.01	8.7	0.7	<0.01
Zn	53	115	73	40	213	66	NS

ESM 8. Minimum, maximum and median of metal concentrations ($mg \cdot kg^{-1}$) in shoots and roots of *Callitriche cophocarpa* from more polluted rivers. P for the Wilcoxon test for comparison of metal concentrations between shoots with roots. NS=not significant

	Shoots			Roots			
	Minimum	Maximum	Median	Minimum	Maximum	Median	p
Cd	0.03	1.1	0.2	0.05	1.8	0.3	<0.01
Co	0.5	14	4.2	4.6	44	13	<0.01
Cr	0.2	22	5.4	2.1	43	3.7	NS
Cu	11	27	21	9.5	29	20	NS
Fe	751	7392	3585	1922	17824	6445	<0.01
Mn	1452	11688	5413	2295	47975	9685	<0.01
Ni	3.9	17	6.5	6.4	22	11	<0.01
Pb	0.5	11	2.0	1.3	28	5.8	<0.01
Zn	71	247	137	75	347	193	<0.01

ESM 9. Minimum, maximum and median of the Bioaccumulation Factor (ratio of metal concentration in shoots and water) for metals in *Callitriche cophocarpa* from clean and polluted sites

	Clean			Polluted		
	Minimum	Maximum	Median	Minimum	Maximum	Median
Cd	0.0002	0.02	0.004	0.0003	0.01	0.004
Co	0.002	0.04	0.02	0.002	0.1	0.01
Cr	0.001	0.1	0.01	0.0001	0.1	0.02
Cu	0.02	0.02	0.01	0.003	0.05	0.02
Fe	1.1	9.7	6.3	5.1	57	7.7
Mn	5.8	112	33	18	91	45
Ni	0.001	0.03	0.002	0.001	0.01	0.002
Pb	0.002	0.2	0.1	0.001	0.02	0.01
Zn	0.02	0.1	0.04	0.01	0.1	0.04

ESM 10. Minimum, maximum and median of the Bioaccumulation Factor (ratio of metal concentration in roots and water) for metals in *Callitriche cophocarpa* from clean and polluted sites

	Clean			Polluted		
	Minimum	Maximum	Median	Minimum	Maximum	Median
Cd	0.001	0.1	0.01	0.001	0.03	0.01
Co	0.01	0.1	0.02	0.002	0.02	0.01
Cr	0.01	0.2	0.02	0.001	0.2	0.01
Cu	0.003	0.02	0.01	0.003	0.06	0.02
Fe	6.1	29	15	9.1	137	25
Mn	17	311	72	24	362	111
Ni	0.00002	0.02	0.002	0.002	0.01	0.004
Pb	0.003	0.07	0.05	0.01	0.1	0.02
Zn	0.02	0.3	0.03	0.01	0.13	0.06
